# Consensus Gene Co-Expression Network Analysis Identifies Novel Genes Associated with Severity of Fibrotic Lung Disease

**DOI:** 10.3390/ijms23105447

**Published:** 2022-05-13

**Authors:** Sudhir Ghandikota, Mihika Sharma, Harshavardhana H. Ediga, Satish K. Madala, Anil G. Jegga

**Affiliations:** 1 Division of Biomedical Informatics, Cincinnati Children’s Hospital Medical Center, Cincinnati, OH 45229, USA; sudhir.ghandikota@cchmc.org (S.G.); mihika.sharma@cchmc.org (M.S.); 2 Department of Electrical Engineering and Computer Science, University of Cincinnati, Cincinnati, OH 45221, USA; 3 Division of Pulmonary Medicine, Cincinnati Children’s Hospital Medical Center, Cincinnati, OH 45229, USA; harshavardhana.ediga@cchmc.org; 4 Department of Biochemistry, National Institute of Nutrition, Hyderabad 500 007, India; 5 Department of Pediatrics, University of Cincinnati College of Medicine, Cincinnati, OH 45267, USA

**Keywords:** Idiopathic pulmonary fibrosis, weighted gene co-expression network analysis, lung fibrosis, gene modules, consensus network analysis, CRABP2

## Abstract

Idiopathic pulmonary fibrosis (IPF) is a severe fibrotic lung disease characterized by irreversible scarring of the lung parenchyma leading to dyspnea, progressive decline in lung function, and respiratory failure. We analyzed lung transcriptomic data from independent IPF cohorts using weighted gene co-expression network analysis (WGCNA) to identify gene modules based on their preservation status in these cohorts. The consensus gene modules were characterized by leveraging existing clinical and molecular data such as lung function, biological processes, pathways, and lung cell types. From a total of 32 consensus gene modules identified, two modules were found to be significantly correlated with the disease, lung function, and preserved in other IPF datasets. The upregulated gene module was enriched for extracellular matrix, collagen metabolic process, and BMP signaling while the downregulated module consisted of genes associated with tube morphogenesis, blood vessel development, and cell migration. Using a combination of connectivity-based and trait-based significance measures, we identified and prioritized 103 “hub” genes (including 25 secretory candidate biomarkers) by their similarity to known IPF genetic markers. Our validation studies demonstrate the dysregulated expression of *CRABP2*, a retinol-binding protein, in multiple lung cells of IPF, and its correlation with the decline in lung function.

## 1. Introduction

Idiopathic pulmonary fibrosis (IPF) is a rare lung disease characterized by irreversible scarring of the lung parenchyma leading to dyspnea, progressive decline in lung function, and respiratory failure [1]. Typical clinical course in IPF includes slow and steady loss of lung function resulting in a low median survival rate post disease diagnosis [2]. Both epigenetic and transcriptional changes are associated with IPF risk and clinical phenotype. Existing approaches use imaging and histological features for clinical assessment and IPF diagnosis. Several studies have been able to identify gene signatures using transcriptomic profiles from both lung tissue [3,4,5] and peripheral blood mononuclear (PBMC) [6] samples. While they were able to distinguish IPF samples from the healthy controls, the cellular and molecular basis for the IPF disease progression is still relatively unknown.

The established marginal statistical analysis methods tend to ignore the interactions between the genes and often result in gene lists that are functionally incoherent with no unifying biological theme [7]. Network-based methods on the other hand amount to pathway-based gene selection approaches [8] as they consider the strength of interactions and intramodular connectivity between the genes and are therefore known to produce biologically meaningful gene lists. Recent attempts have been made to analyze gene co-expression networks to detect key genes, pathways, and regulatory factors in IPF [9,10,11,12,13,14]. These modules are often strongly enriched for distinct functional categories, phenotype-genotype associations, or cell type markers. However, existing studies in IPF typically focus on a single cohort and do not leverage all the transcriptomic data available. Consensus mechanisms that combine multiple networks could lead to the identification of gene sets or pathways that are strongly conserved between studies involving related but different diseases or different biological tissues within the same disease. Results based on such consensus unsupervised approaches are hypothesized to be more robust and biologically relevant when compared to those from a single dataset or cohort.

In this study, we implemented a consensus version of weighted gene co-expression network analysis (WGCNA) on two lung transcriptomic cohorts to identify conserved clusters of genes. Candidate modules were identified based on their correlations with IPF phenotypic traits, including the forced vital capacity (FVC) and diffusing capacity of lungs for carbon monoxide (DL_CO_). Highly connected “hub” genes within the modules were retrieved based on the strength of their associations with the phenotypic traits. We further characterized and prioritized these modules by leveraging (i) single-cell RNA-seq-based cell markers from normal and IPF lung samples, and (ii) genome-wide association studies (GWAS) based on lung function traits and gene associations. Additionally, the hub genes within these modules were filtered using curated gene lists to obtain novel fibrotic candidates and potential biomarkers. The final set of secreted proteins and novel candidate genes were validated with the help of independent lung transcriptomic datasets. These candidate biomarkers were also found to distinguish and categorize IPF and other respiratory lung disorders.

## 2. Results

### 2.1. Consensus Gene Modules

**Figure 1:**
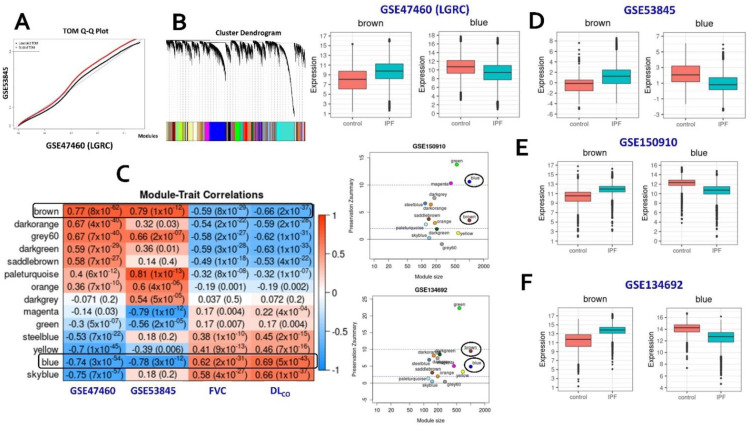
Consensus WGCNA steps. (**A**) Q–Q plot of the scaled TOM matrices of both the training cohorts. (**B**) Hierarchical dendrogram constructed based on the consensus TOM matrix. (**C**) Correlation heatmap of consensus modules with the phenotypic traits from both training datasets. Candidate modules identified based on the correlation strength with phenotype status in both cohorts (GSE47460 or LGRC; GSE53845) and the lung-function traits (FVC and DL_CO_) from the LGRC cohort. Only modules with a disease (IPF) correlation ≥0.5 in either of the two training cohorts have been included in the heatmap. (**D**) Box plots illustrating the expression levels of intramodular hubs from the two candidate modules (brown and blue) in the two cohorts. (**E**,**F**) Scatter plots of module preservation scores of consensus modules (on the left) and box plots of expression levels of the brown and the blue modules (on the right) in two independent validation cohorts, namely, GSE150910 (Panel **E**) and GSE134692 (Panel **F**).

Normalized gene expression profiles from whole lung tissues across the two training cohorts (GSE47460 and GSE53845) were extracted from the NCBI GEO website. The LTRC cohort (GSE47460) was filtered to retain only the IPF samples and controls. Using the expression profiles of 15,180 genes found in both studies as inputs, we identified 32 consensus gene modules (Figure 1A,B and Supplementary Figure S1b) (Supplementary Methods). From these 32 modules, we selected 14 modules that showed a significant correlation (correlation ≥ 0.5; *p*-value < 0.05) with the disease in at least one cohort (Figure 1C). Genes within these modules are hypothesized to be co-expressed in samples from both studies [15]. The sizes of these consensus modules were in the range of 100 to 2000 genes (Supplementary Table S4). All the pre-processing, and analysis steps, were implemented using the WGCNA R package [16].

### 2.2. Identification of Conserved Candidate Modules Correlated with IPF Phenotypic Traits

Candidate modules that are significantly correlated with phenotypic traits in addition to being strongly preserved in independent validation studies are hypothesized to represent gene clusters associated with distinct molecular mechanisms of the investigated disease. To identify candidate modules associated with IPF, the consensus gene modules were first correlated with the sample phenotype information (IPF or Control) using the module eigengenes (Supplementary Methods). Additionally, the FVC and DL_CO_ lung function traits from the LGRC cohort (GSE47460) were used in the analysis. We found three consensus modules significantly correlated (|Cor|≥0.5;Pval (adj)≤0.05) with both the phenotype status and the lung function traits (Figure 1C). The brown (1333 genes) and grey60 (305 genes) modules were significantly upregulated in IPF patients in both cohorts while being significantly associated with DL_CO_ and FVC. Similarly, the blue (2020 genes) module was observed to be downregulated in IPF samples from both cohorts (Figure 1D). Statistical significance of these module correlations was assessed using Fisher’s asymptotic *p*-values.

Next, we implemented module preservation analysis to obtain the preservation status ($Z_{summary})$ of all consensus modules (Supplementary Methods). We used two independent datasets (GSE150910 and GSE134692) as the test networks and alternated the two training datasets as reference networks. Based on the composite Z scores, we observed that the brown and blue modules were preserved in both the test cohorts (Supplementary Figure S2a,b). The brown module was moderately preserved in both the datasets while the downregulated blue module was preserved strongly in GSE150910 (Figure 1E and Supplementary Figure S2a) and moderately in GSE134692 (Figure 1F and Supplementary Figure S2b). On the other hand, the grey60 module, which was correlated strongly with IPF and lung function traits, was found to be poorly preserved in both the test networks and was therefore not considered for additional downstream analyses.

Genes within each module were ordered based on their HubScore (see Supplementary Methods), and the top 5% of them, retrieved based on the 95th percentile threshold scores, were categorized as the “intramodular hubs”. There was a total of 170 hub genes from the two candidate modules with 68 of them upregulated (brown module) and 102 downregulated genes (blue module) in IPF (Supplementary Table S4). These 170 hub genes were able to categorize and distinguish the IPF patients from the healthy controls in both the training cohorts (Supplementary Figures S3a,b) and in independent datasets, used for validation (Supplementary Figure S4). We further ranked these hub genes based on their similarities to known GWA IPF genes and intersected them with secreted proteins to identify secreted biomarkers and novel IPF-related genes. They were further used to characterize the two phenotype correlated consensus modules. Finally, a filtered set of these hub genes were found to distinguish IPF samples from those of other interstitial lung diseases.

### 2.3. Consensus Modules-Biological Processes

**Figure 2:**
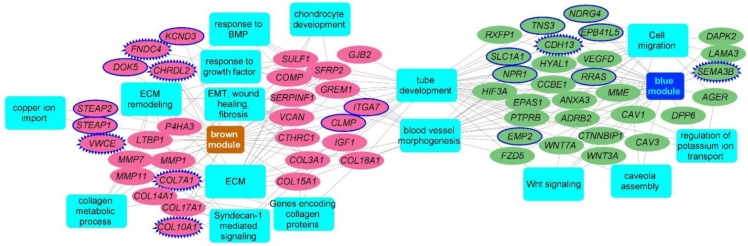
Functional enrichment analysis of hub genes from brown and blue modules. Network representation of select enriched biological processes and pathways among the intramodular hubs from the brown and the blue candidate modules. An edge between a gene (oval node) and a functional annotation (rectangular node) indicates the membership of that gene to that annotation term. Enriched terms are represented as blue color rectangles. Upregulated hub genes of brown module are in pink color while the downregulated genes from the blue module are in green color. Novel fibrotic candidates are highlighted using blue color solid node borders while the secreted novel hubs are highlighted with zig-zag blue borders. Network is generated using Cytoscape application.

For functional characterization of the gene modules, we used the intramodular hubs from each of the candidate modules as inputs to the ToppFun application of the ToppGene Suite [17]. The brown module, upregulated in IPF, was expectedly enriched for the extracellular matrix (ECM) organization (15 genes; FDR B&H: 1.36 × 10^−10^), genes encoding ECM and ECM-associated proteins (22 genes; 1.36 × 10^−9^), and biological processes related to ECM organization (16 genes; FDR B&H: 4.76 × 10^−10^). It was also enriched for genes encoding collagen proteins, collagen formation, and collagen metabolic processes. Additionally, the brown module also consisted of genes involved in EMT, wound repair, and fibrosis (7 genes; FDR B&H: 3.48 × 10^−2^), BMP signaling (5 genes; FDR B&H: 2.74 × 10^−2^), and chondrocyte development and differentiation (4 genes; FDR B&H: 2.40 × 10^−2^). Hubs from the downregulated blue module were enriched for tube morphogenesis (21 genes; FDR B&H: 5.33 × 10^−5^) and tube development (24 genes; 3.63 × 10^−5^), blood vessel morphogenesis (16 genes; 6.15 × 10^−4^), regulation of cell motility (15 genes: FDR B&H: 2.86 × 10^−2^), and epithelial cell migration (9 genes; FDR B&H: 1.40 × 10^−2^) (Figure 2) (Supplementary Table S5).

### 2.4. Consensus Modules for Specific Cell Types from Normal, and Fibrotic Lung Markers

We next used multiple scRNA-seq studies, and their reported lung cell-type-based markers to detect the specific cell-type identities of the candidate modules. We performed enrichment analysis against the compiled cell type markers from scRNA-seq studies of both normal and IPF lung tissues. Expectedly, the brown module hubs were enriched for genes expressed in fibroblasts and other mesenchymal cell types (Supplementary Table S6). Specifically, significant enrichment of myofibroblast markers was observed in both normal lung (6 genes) and IPF lung (11 genes). The brown module hubs also included several adventitial fibroblast markers (18 genes). Accordingly, we found several intramodular hubs (*DCLK1, COMP, SFRP2, LTBP1, SULF1, CRABP2, CTHRC1, COL3A1, FHL2, COL15A1, COL18A1, CLMP, COL14A1, TSHZ2, VCAN,* and *SERPINF1*) in the brown module that were upregulated in IPF lung myofibroblast cells [18] when compared to those from healthy controls (Supplementary Table S6). Interestingly, a recent study has shown that *GPX8* expression is upregulated during the epithelial–mesenchymal transition (EMT) program and that loss of *GPX8* confers epithelial characteristics in the mesenchymal cell lines [19]. Likewise, *DCLK1* is known to promote EMT [19]. Recent studies have suggested that EMT may promote a pro-fibrotic microenvironment by dysregulating paracrine signaling between epithelial and mesenchymal cells and the therapeutic potential of targeting EMT in fibrotic conditions [20].

The blue module on the other hand contained a significant number of marker genes expressed in alveolar epithelial type 1 (AT1) cells in both healthy and fibrotic human lung tissue. Interestingly, we also found several genes (*VSIG10, HSD17B6, N4BP1, PLLP, HPCAL1, MYRF, GPM6A,* and *AGER*) that are downregulated in AT1 cells from IPF lung tissue [18] relative to healthy samples. On the other hand, markers of alveolar fibroblast cells were found to be downregulated in IPF and enriched (17 genes) in the downregulated blue module. One of the enriched genes includes the glutamate receptor *GRIA1*, a unique canonical marker of the newly identified alveolar fibroblast cells [21]. These cells were found to be involved in the recruitment of immune cells and the complement system. Significant enrichment of AT2-signaling (7 genes) [21] and transitional AT2 (9 genes) [22] markers were also observed (Supplementary Table S6).

### 2.5. Candidate Biomarkers and Novel IPF-Associated Genes

To identify potential candidate biomarkers for IPF, the disease-related hub genes were intersected with a pre-compiled list of secreted proteins (Supplementary Table S3) from the human protein atlas (HPA) and 57 candidate biomarkers were identified (Supplementary Table S4). These 57 secreted hubs were able to differentiate the IPF patients from healthy controls in both the training cohorts (Supplementary Figure S3c,d) as well as independent validation datasets (Supplementary Figure S5). Of the 57 genes, 30 genes were in the upregulated brown module and 27 genes were found in the downregulated blue module. Furthermore, 12 of these are candidate secretory biomarkers (*CFB, CFI, EFNA4, GREM1, IGF1, SCG5, SERPINF1, VCAN, HYAL1, KL, PCDH12,* and *VEGFD*) (Supplementary Table S3). Moreover, 12 genes from this secreted list (*IGF1, LTBP1, SULF1, SFRP2, COL15A1, MMP1, CFI, COL3A1, AGER, WNT7A, CDH13,* and *CRTAC1*) are potentially associated with pulmonary fibrosis.

**Figure 3:**
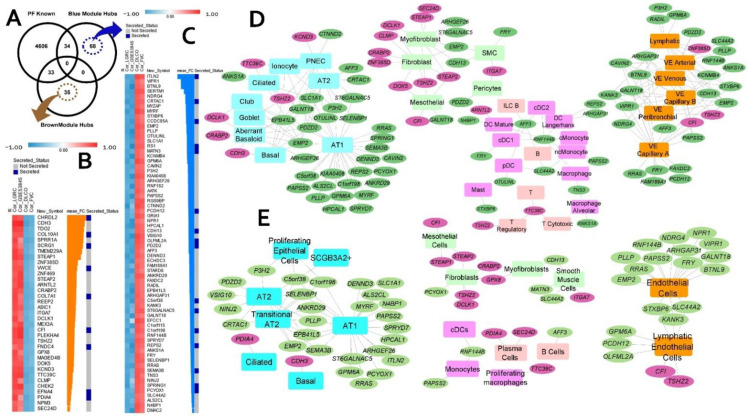
Novel IPF candidate genes and their cell types (based on two IPF scRNA-seq datasets). (**A**) Venn diagram illustrating the overlap between intramodular hubs with a compiled set of known pulmonary fibrosis genes. (**B**,**C**) Heatmap representation of the IPF and lung function (FVC and DL_CO_) correlations of the 35 and 68 novel candidate genes from brown and blue modules, respectively. Also shown in the heatmap are their mean fold change in IPF and whether they encode known secretory proteins. Heatmaps were generated using the Morpheus application. Panels (**D**,**E**) represent the lung cell associations for the novel candidate genes using two different single-cell data sets from IPF. The pink and green ellipses represent genes from brown (upregulated in IPF) and blue (downregulated in IPF) modules, respectively. The rectangular nodes are different cell types. The cell types are color coded based on their broad cell type, namely, blue for epithelial, light green for mesenchymal, light, and deep pink for lymphoid- and myeloid-immune, respectively, and orange for endothelial cell types. Networks are generated using Cytoscape application.

To identify potential novel fibrotic candidates, we have compiled a set of more than 4600 fibrosis-related genes (Supplementary Table S1) from several sources (see Section 4). Intersecting these genes with the consensus module hub genes resulted in 103 potentially novel candidates (35 upregulated, 68 downregulated in IPF) (Figure 3A) that are previously not associated with pulmonary fibrosis (PF). Each of these 103 novel candidates is strongly correlated with IPF (two independent cohorts) and lung function (Figure 3B,C). Additionally, among these 103 candidate genes, there are at least 25 genes known to encode secretory proteins (Figure 3B,C) suggesting their potential as novel biomarkers in IPF. Several of these 103 novel candidates are found to be differentially expressed (cell marker genes) in different lung cell types (Figure 3D,E).

Out of the 35 upregulated novel hub genes from the brown module, 14 candidates are marker genes in at least one of the lung single cells studies used in our analysis. A total of 10 upregulated novel hubs were found amongst the markers from multiple mesenchymal cell types, including 8 markers genes (*STEAP2, STEAP1, TSHZ2, DCLK1, CLMP, SEC24D, CRABP2,* and *GPX8*) in myofibroblasts. Moreover, *DCLK1, CRABP2, CLMP*, and *TSHZ2* are overexpressed in myofibroblast cells of the fibrotic lung [18,22]. Some of the novel candidates (*TTC39C, CDH3, TSHZ2, CRABP2, DCLK1,* and *PDIA4*) from the brown module are simultaneously expressed in lung epithelial cells. Interestingly, three of the novel hubs, *CRABP2*, *CDH3*, and *DCLK1* are found to be expressed in aberrant basaloid cells, a novel epithelial cell population that co-express basal epithelial markers and are located at the edge of myofibroblast loci in the IPF lung [18,22] (Figure 3D,E).

Similarly, several of the 68 novel candidates from the downregulated blue module are marker genes in normal or IPF lung, with most of them (30 genes) expressed in alveolar epithelial type 1 (AT1) cells (Figure 3D,E). We observed eleven genes expressed in AT2 cells. Additionally, nine more candidates (*ANKRD29, C1orf198, C5orf38, CRTAC1, EMP2, EPB41L5, PLLP, SELENBP1,* and *SEMA3B*) were reported to be expressed in transitional AT2 cells in IPF lung tissue [22]. Interestingly, a recent study reported that the CRTAC1 protein levels in lung lavage fluid and blood plasma is a novel peripheral protein biomarker of the lung alveolar epithelial health status reflecting the de-differentiation of AT2 cells in lung fibrosis [23]. Genes expressed in AT2-signaling cells are found to be involved in Wnt signaling and detoxification while the transitional AT2 cells potentially represent a state during the differentiation trajectory from AT2 to AT1. Several of the downregulated candidates (*GRIA1, EFCC1, CAVIN2, OLFML2A, CDH13, EMP2, PAPSS2, ECHDC3,* and *KCNMB4*) are overexpressed in alveolar fibroblast cells in normal lung. Finally, we also observed a significant number of novel candidates (*FAXDC2, EMP2, PLLP, HPCAL1, GPM6A, PAPSS2, RRAS, GALNT18, AFF3, ANKS1A, CAVIN2, P3H2,* and *PDZD2*) expressed in both epithelial and vascular-endothelial cells of human lung tissue (Supplementary Table S6).

### 2.6. Consensus Hubs Associated with Lung Function Activity

**Figure 4:**
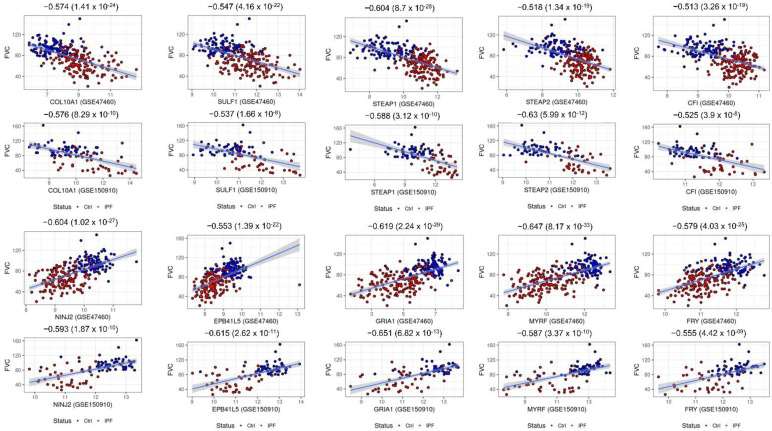
Scatter plots of select candidate hub genes associated with lung function (FVC). Each plot includes sample FVC measures as a function of expression values of intramodular hub genes. All the plots are annotated by the correlation values between the sample FVC measure and the sample expression profile for each gene. Scatter plots for five genes each from the brown (*COL10A1*, *SULF1*, *STEAP1*, *STEAP2*, and *CFI*) and blue (*NINJ2*, *EPB41L5*, *GRIA1*, *MYRF*, and *FRY*) modules and their FVC correlations from two independent IPF cohorts are shown. Student’s asymptotic *p*-value for given correlations is also included.

Loss of lung function activity is a prominent symptom in IPF resulting in a low median survival rate post diagnosis. To find if any of the IPF-related hubs are associated with lung function, we used the GWA gene set associated with the lung function traits (Section 4). Intersecting them with the intramodular hubs from both candidate modules resulted in 59 lung function-associated genes. The upregulated brown module consisted of 22 lung function candidates, including 12 potential novel candidates (*CFI, COL10A1, PDIA4, SCRG1, ASIC1, GPX8, STEAP2, STEAP1, TTC39C, ITGA7, ZNF385D,* and *DCLK1*). Both *CFI* and *DCLK1* are marker genes of several mesenchymal cells like adventitial fibroblasts and lipofibroblasts. Furthermore, the *CFI* gene was found to be expressed in mesothelial cells while *DCLK1* is a marker gene of the newly identified aberrant basaloid cells [18]. Moreover, *CFI, COL10A1, PDIA4,* and *SCRG1* are all found to be secreted. The blue module contained 37 IPF downregulated genes which are known to be associated with lung function traits. Of those, 24 novel candidate genes (*AFF3, NINJ2, FRY, ARHGAP31, ARHGEF26, ANKS1A, EPB41L5, EMP2, SPRYD7, SLC44A2, MYRF, KCNMB4, C1orf115, ECHDC3, RNF144B, CTNND2, GRIA1, DENND3, CCDC85A, CDH13, MATN3, OLFML2A, PCYOX1,* and *SEMA3B*) were identified (Supplementary Table S2). Most of them were found to be expressed in multiple epithelial and vascular endothelial cell populations of both normal and fibrotic lung samples. Each of these lung function candidates is significantly correlated with DL_CO_ and/or FVC sample measurements (data from both GSE47460 and GSE150910 cohorts) (Table 1 and Figure 4). Thus, apart from identifying genes related to lung function in IPF, our approach was able to provide additional GWAS-based genetic evidence to some of the novel candidates identified earlier.

### 2.7. Hub Genes Conserved across Different IPF Severities and Acute Exacerbation

**Figure 5:**
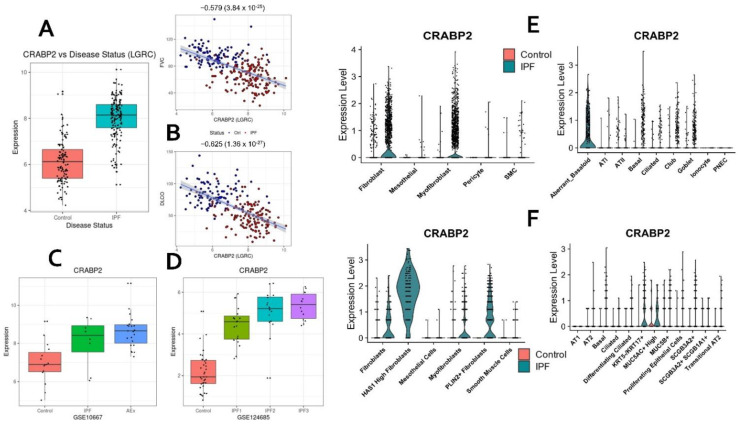
*CRABP2* as a novel candidate for IPF. *CRABP2* is significantly upregulated in IPF (**A**) and is negatively correlated with lung function (FVC and DL_CO_; **B**). Additionally, *CRABP2* is differentially expressed in acute exacerbation (**C**) and shows a gradient level upregulation in three stages of IPF, namely, early (IPF1), progressive (IPF2), and advanced (IPF3) stages (**D**). *CRABP2* is enriched in both epithelial (aberrant basaloid or KRT5-/KRT17+ and goblet cells) and mesenchymal (fibroblast, myofibroblast, HAS1-high fibroblasts, and PLIN2+ fibroblasts) cells (**E**,**F**).

To identify candidate hubs differentially expressed across different IPF severities, we used a recently published lung transcriptomic study (GSE124685) [25] that used quantitative micro-CT imaging and tissue histology on IPF lung samples to categorize IPF stages (IPF1 or early-stage IPF, IPF2 or progressive stage IPF, and IPF3 or end-stage IPF) representing the increasing extent of fibrotic remodeling, lower alveolar surface density (ASD) and higher collagen content. We first performed module preservation analysis to observe the preservation status of both the candidate modules in the different IPF subtypes. In our experiments, we observed that both the blue and brown gene modules were strongly preserved in all the three sample sets (Supplementary Figure S6). Next, we extracted the differentially expressed gene sets (DEGs) from each of the IPF severity levels (early, progressive, and end-stage) using the limma approach [26]. Intersecting the DEGs from IPF1, IPF2, or IPF3 stages with the consensus hubs resulted in an overlap of 51/68 (75%) upregulated (brown module) and 76/102 (~75%) downregulated (blue module) hubs with any of the three stages in IPF. A substantial number (41 brown module hub genes and 30 blue module hub genes) were differentially expressed in all three stages of IPF (Supplementary Table S7). Of the thirty-five novel candidate hub genes from the brown module, 14 genes (*CDH3, CFI, CHRDL2, COL10A1, CRABP2, DCLK1, DOK5, FNDC4, GPX8, SCRG1, SPRR1A, STEAP1, STEAP2,* and *TDO2*) were found to be overexpressed in all the three IPF stages (Table 2). Among these, *CDH3, CRABP2,* and *DCLK1* are expressed in aberrant basaloid cells while *CRABP2, DCLK1, DOK5, GPX8, STEAP1,* and *STEAP2* are expressed in myofibroblasts or fibroblasts suggesting early upregulation in the progression of the disease. Several of these genes (e.g., *CRABP2*) are strongly correlated with lung function, differentially expressed in acute exacerbation, and showed a gradual increase in expression from IPF1 to IPF3 (Figure 5A–D). *CRABP2* is also expressed in IPF-specific epithelial (aberrant basaloid cells) and IPF-specific mesenchymal cell (HAS1 high and PLIN2+ fibroblasts) populations (Figure 5E,F). Similarly, 30/68 novel hubs from the blue module were downregulated in all three IPF stages (Table 2). These included four genes (*EMP2, EPB41L5, PLLP, CRTAC1*) from transitional AT2 cells and the translational utility of *CRTAC1* as an IPF biomarker [23] has been reported recently.

Similarly, we identified several intramodular hub genes differentially expressed in the acute exacerbation of the IPF phenotype (IPF-AEx). Specifically, we found 54/68 upregulated hubs (FDR *p*-value = 2.04 × 10^−61^) including 27/35 novel candidates (FDR *p*-value = 2.01 × 10^−30^) that are overexpressed in the IPF-AEx tissue samples (Supplementary Table S7). Among the blue module hubs, 46/102 genes (FDR *p*-value = 1.83 × 10^−56^) were downregulated in the IPF-AEx phenotype with 26/68 novel candidates (FDR *p*-value = 9.07 × 10^−30^) among them (Supplementary Table S7). Interestingly, we found 8 novel candidate genes (*FRY, SEMA3B, SLC1A1, C5orf38, NINJ2, VSIG10, PDZD2,* and *SELENBP1*) from the blue module (FDR *p*-value = 1.02 × 10^−6^) that were downregulated exclusively in IPF lung tissue samples with acute exacerbations. Differentially expressed genes in IPF-AEx were obtained from a previously published study [27] using the limma approach.

### 2.8. Candidate Genes Categorizing IPF and Other Interstitial Lung Diseases

Next, we investigated whether the candidate hub genes we have identified can differentiate IPF from other interstitial lung disorders (ILDs). To do this, we developed and trained regularized logistic regression models [28] using novel hub genes as independent predictors and evaluated them on test samples from independent test cohorts or test partitions from within the LGRC study (see Supplementary Methods). The elastic net regularization method linearly combines both L1 and L2 regularization schemes with a mixing parameter α and is useful for effective variable selection [23]. Firstly, we identified 42 novel hubs (α=0.35) that were able to categorize the IPF samples with a precision-recall area under curve (PRAUC) scores of 0.923 (FDR *p*-value = 0.05) and 0.98 (FDR *p*-value = 0.05) in the two test studies (GSE150910 and GSE134692) (Supplementary Figure S7a). Among them were eleven secreted proteins—*MATN3, C12orf49, COL7A1, COL10A1, EFNA4, ST6GALNAC5, C5orf38, CRTAC1, VWCE, CFI*, and *PDZD2* (Table 3). On the other hand, the model with the complete set of novel candidates (α=0) as predictors did not show any significant improvements in terms of PRAUC scores (0.937 in GSE150910 and 0.967 in GSE134692). As mentioned previously, a recent study reported CRTAC1 protein levels in lung lavage fluid and blood plasma as a novel peripheral protein biomarker of the lung alveolar epithelial health status [23], indicating the translational utility of our findings. Next, we identified 45 marker genes (α=0.2) capable of distinguishing IPF from chronic hypersensitive pneumonitis (CHP) samples (from GSE150910) with a PRAUC score of 0.804 (FDR *p*-value = 0.0077; Supplementary Figure S7b). Several of these filtered candidates were found to be secreted (*C12orf49, COL10A1, FNDC4, ITLN2, MATN3, PDZD2, RS1, SCRG1,* and *ST6GALNAC5*) making them potential candidate biomarkers useful for distinguishing IPF from pneumonitis. In comparison, the full model containing all 170 intramodular hubs as independent variables underperformed (PRAUC = 0.785; FDR *p*-value = 0.02) (Table 3).

### 2.9. Hub Gene Prioritization

Novel intramodular hub genes from the brown (35 genes) and blue (68 genes) modules were further prioritized based on their functional relatedness (*guilt by association*) to a set of known IPF genes. We compiled and used a list of 34 GWA genes from GWAS Catalog [29] as the training set (Supplementary Table S1). The ToppGene application from the ToppGene Suite [17] was used to compute the functional similarity scores between the training set and each of the two intramodular hub gene sets separately (Table 4; Supplementary Table S8). The hub genes are ranked based on their similarity to the training set genes (34 IPF GWA genes) in gene ontology (GO), both mouse and human phenotype ontologies, biological pathways, diseases, and single-cell gene annotations. The top twenty ranked genes from brown and blue modules are listed in Table 4.

### 2.10. CRABP2—Novel Candidate Gene and Potential Biomarker of IPF

As a proof-of-concept to demonstrate the translational relevance of our study, we selected cellular retinoic acid-binding protein 2 (*CRABP2)*, one of the novel intramodular hubs that is (a) upregulated in IPF lungs; (b) differentially expressed across different IPF severity levels; (c) part of the novel candidate genes that are capable of distinguishing fibrotic samples from healthy controls in the two training cohorts and two other independent validation studies (GSE150910 and GSE134692); (d) shows cell-specific expression; and (e) encodes a secretory protein (Figure 5). CRABP2 is an intracellular lipid-binding protein associated with retinoic acid and modulates retinoic acid signaling in the cell [30].

**Figure 6:**
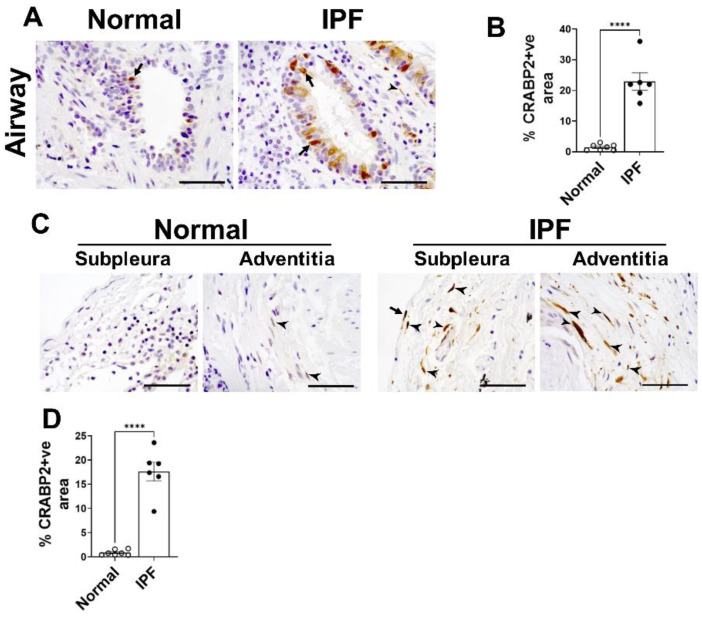
Upregulation of CRABP2 in the lungs of IPF. Immunostaining was performed using antibody against CRABP2 on the lung sections of normal and IPF. (**A**) Representative images of distal lung airway from normal and IPF lungs immunostained with anti-CRABP2 antibody and images were taken at 60× magnification. Scale bar: 50 µm. Arrows are used to highlight airway epithelial cells positive for CRABP2. (**B**) Quantification of the percent of CRABP2 staining in airways in the total lung area of images analyzed using BZ-X analyzer. (**C**) Representative images of subpleural and adventitia from normal and IPF lungs immunostained with anti-CRABP2 antibody and images were taken at 60× magnification. Scale bar: 50 µm. Arrows and arrow heads are used to highlight mesothelial cells lining the lung and spindle-shaped fibroblasts, respectively. (**D**) Quantification of the percent of CRABP2 staining in subpleural and adventitial area in the total lung area of images analyzed using BZ-X analyzer. (**** *p* < 0.00005, *n* = 6–7/group; Student’s *t*-test).

To validate whether upregulation of *CRABP2* is also reflected in protein levels in IPF, we immunostained lung sections of IPF and control lungs using CRABP2-specific antibody and quantified CRABP2 levels in both subpleural and distal airways of the lungs. Notably, we observed a significant increase in immunostaining of CRABP2 in both airway epithelial cell types and spindle-shaped fibroblasts in the distal fibrotic lung lesions of IPF compared to control lungs (Figure 6). While our preliminary results are promising regarding the feasibility and utility of *CRABP2* as a novel diagnostic candidate gene in IPF, further investigation is warranted to determine whether observed increases in CRABP2 levels associate with the disease progression and severity of fibrosis in preclinical models of pulmonary fibrosis.

## 3. Discussion

Leveraging multiple transcriptomic datasets, we implemented a network-based approach to identify consensus co-expressed gene clusters associated with IPF phenotypes, biological processes, and pathways implicated in IPF onset and progression. Using lung transcriptomic profiles of IPF patients and healthy controls from two independent cohorts (GSE47460 and GSE53845), we identified thirty-two consensus gene modules. Of these, we prioritized two candidate modules (brown and blue) based on their correlations with FVC and DL_CO_ lung function traits, phenotype status and their level of preservation in two independent test datasets (GSE150910 and GSE134692). Interestingly, these modules consisted of 6 GWA genes (*ABCA3, OBFC1, TOLLIP, SFTPC, ATP11A,* and *DEPTOR*) reported to be associated with IPF [29].

Using a novel hub score measure based on a combination of connectivity-based and trait-based significance measures enabled us to identify 170 hub genes (68 upregulated, 102 downregulated) that are central in both the candidate modules and are strongly correlated with lung function traits. Selecting only the intramodular hub genes from disease-related consensus modules has been shown to be useful to make the gene lists manageable and with a unifying biological theme [8]. When compared to differentially expressed gene lists, this approach has the advantage of identifying genes that are potentially relevant to the IPF phenotype because it considers lung function measurements when prioritizing the genes. From these intramodular hub genes, we identified 57 secreted candidate biomarkers, 103 novel candidates that are previously not known to be associated with pulmonary fibrosis with 36 lung function-associated genes among them. A majority of the intramodular hub genes were found to be differentially expressed among the various stages of IPF [25] and IPF acute exacerbation. Furthermore, using cell marker genes from both normal and fibrotic lung scRNA-seq studies, we identified 122 hub genes and 68 novel candidates which are marker genes in various single-cell populations in the lung. We also identified sixteen potential candidate biomarkers that are not only capable of classifying IPF samples (versus controls) but also distinguish IPF from other interstitial lung disorders. In all our experiments, these filtered genes have either matched or outperformed the models with complete gene sets as predictors. Further validation studies centered around these hubs could lead to a better understanding of the underlying biological mechanisms, disease progression, and novel therapeutic discovery in IPF.

In comparison to previous studies that have used network analysis for IPF transcriptomic data, our approach has the advantage of using data from two independent cohorts. We also validated the preservation status of the identified gene modules in two independent test cohorts. Another advantage of our current approach is the utilization of the intramodular hub genes instead of the entire modules for characterizing the consensus modules. These hub genes were identified using a novel HubScore metric combining both connectivity-based and trait-based correlations. Further, and most importantly, we have leveraged scRNA-seq-based cell markers with the network hubs to characterize and identify the specific cell-type identities associated with the gene modules.

Our approach does hold certain limitations. Firstly, due to the differences between the reference panels used in the two training datasets, we could include only the common set of transcripts, resulting in the exclusion of several genes. This also puts a pragmatic upper bound on the number of transcriptomic datasets that could simultaneously be used in such approaches. Thus, computational frameworks, based on neural network architectures, which utilize all or most of the available gene expression data for a specific disease could prove to be more fruitful. Another important limitation of our study is the use of hard thresholding of the HubScore measure (top 5%) for filtering the intramodular hub genes from within the candidate modules. In addition, the regularized elastic-net logistic regression models suffer from low sample counts. As a result, some of our models are bound to be impacted by the lack of adequate training data. Therefore, carefully designed experimental validations are needed to validate the hypotheses and candidate biomarkers coming out of our study.

A particularly interesting future work in this direction would be to implement unsupervised graph neural network (GNN) frameworks for module detection on gene networks. These methodologies not only exploit the geometric structure (connectivity among the genes) but also make use of multi-dimensional features that can include functional annotation terms, trait/phenotype associations, cell types, and other features. In conclusion, we present an unsupervised community detection framework on gene co-expression networks that leverage clinical features to discover translationally actionable gene modules and targets. Known markers of different cell types, genetic markers from GWA studies, and biological processes and pathways are used to further characterize and prioritize these gene modules.

## 4. Materials and Methods

### 4.1. IPF Transcriptomic Datasets

**Figure 7:**
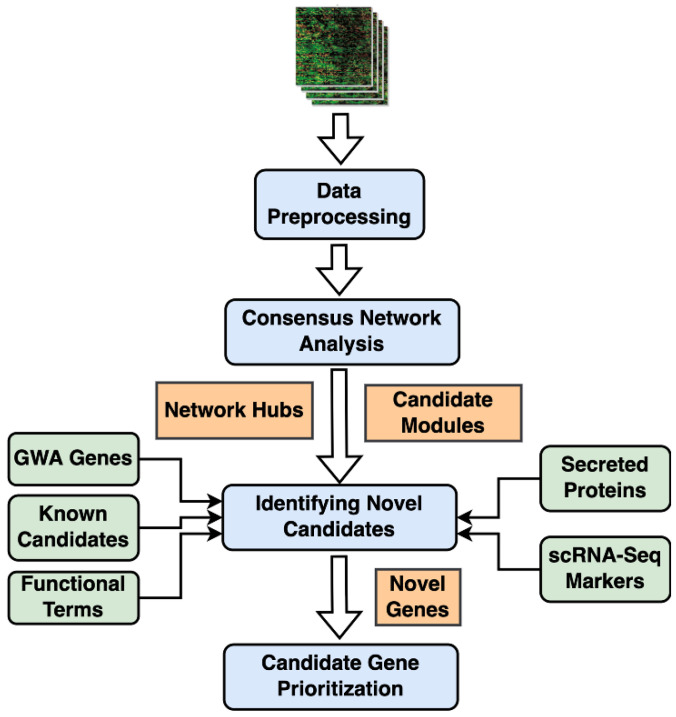
Schematic representation of the workflow: Transcriptomic data from the IPF and controls were downloaded from NCBI’s Gene Expression Omnibus (GEO) and used as inputs for WGCNA consensus Analysis after the necessary data cleaning and normalization steps. Consensus modules obtained from WGCNA were correlated with phenotype status and other IPF-relevant clinical traits to select candidate modules. Intramodular hub genes were identified within each candidate module and used for functional characterization and to identify novel candidates and secreted biomarkers in IPF.

Publicly available microarray lung transcriptomic data were obtained from the NCBI’s Gene Expression Omnibus (GEO) repository [31]. The NHLBI-funded LTRC (Lung Tissue Research Consortium) dataset (GSE47460) contains expression profiles and clinical attributes for 160 IPF patients and 108 healthy controls. Additionally, we included transcriptomic profiles from an independent cohort (GSE53845 [32]) of 40 IPF cases and 8 controls for the consensus analysis (Table 5). Other IPF datasets from the GEO repository were explored but were deemed unsuitable due to the lack of consensus of their topological similarity distributions with the remaining datasets (Figure 7; Supplementary Methods). For validating the candidate modules and novel biomarkers identified, we used two independent cohorts, an RNA-seq study GSE134692 [33] containing 72 lung tissue samples (46 IPF cases and 26 healthy controls) and GSE150910 [24] with 103 tissue samples each from IPF patients and healthy controls. Since different reference platforms are used in these transcriptomic datasets, as part of data pre-processing, we replaced all the probe IDs with their corresponding gene symbols. If multiple transcripts (i.e., probe IDs) were found to be mapped to the same gene, the probe set (along with its mapped gene) with the highest expression value was retained. All the expression datasets were also checked for genes and samples with excessive missing values.

### 4.2. Normal and IPF Lung Single-Cell Markers

For filtering out novel IPF and lung function-related genes from the modules, we compiled single-cell transcriptomic signatures in both normal [21,22] and IPF lung samples [18,22,34]. Only significant associations (FDR *p*-value ≤ 0.05; log fold change or FC ≥ 0.5) were retained in the analysis. In the case of IPF signatures, the upregulated and downregulated genes were distinguished based on the fold change values and used separately for enrichment analysis. Similarly, human lung single-cell markers from two different protocols [21] were used separately during the enrichment step.

### 4.3. Known Pulmonary Fibrosis Genes

In addition to markers from individual cell types in lung tissues, several curated gene sets were also used in the post-processing of the disease-related gene modules. A list of 4673 known pulmonary fibrosis genes was compiled from literature and different databases (Supplementary Table S1). This list comprised human genes associated with “Pulmonary fibrosis”, “Idiopathic pulmonary fibrosis”, and “Interstitial Lung Disease” from Open Targets platform [35], CTD [36], Phenopedia [37], and GeneCards [38] databases along with a literature search on PubMed. This compiled list of 4673 pulmonary fibrosis genes was used to obtain novel candidate genes for pulmonary fibrosis.

### 4.4. Hub Gene Prioritization

Intramodular hub genes from phenotype correlated consensus modules were ranked based on their functional similarity to a set of known IPF genes. We compiled and used a list of 34 GWA genes from GWAS Catalog [29] as the training set (Supplementary Table S1). ToppGene application from the ToppGene Suite [17] was used to further prioritize the candidate genes using the *guilt by association* principle of functional relatedness (Figure 7).

### 4.5. Lung Function GWA Genes

A second set of curated genes include those that are associated with lung function traits (DL_CO_, FVC/FEV) extracted from both Open Targets Genetics [35] and GWAS Catalog [29] portals. These genes are associated based on significant GWAS variants (*p*-value < 10^−5^) mapped from multiple studies. At the time of drafting this manuscript, 5416 such genes were identified from the two portals and utilized to filter out lung function-related genes from the consensus modules (Supplementary Table S2).

### 4.6. Secreted Proteins

The “secretome” was compiled using a list of proteins known to be secreted by cells from the Human Protein Atlas [39] database. This gene set consisting of 2640 genes was used for the biomarker discovery (Supplementary Table S3). It included 742 blood-secreted markers and 234 genes secreted to the extracellular matrix.

### 4.7. Consensus WGCNA and Candidate Modules

A weighted gene co-expression network analysis (WGCNA) framework is used to identify clusters of co-expressed genes [40,41] based on pairwise correlations between gene expression profiles across all the samples (Supplementary Methods). WGCNA consensus module analysis is used to find highly connected genes preserved among multiple datasets [42]. It involves constructing co-expression networks for each dataset and then identifying consensus modules among them.

WGCNA identified modules are ranked and prioritized by correlating them with sample clinical traits and phenotypic status [15]. Disease-related candidate modules are selected based on the strength and statistical significance (Student’s asymptotic *p*-values) of these module correlations with the phenotypic traits (Supplementary Methods). Further, module preservation analyses are used to filter the candidate modules based on their preservation status in other test networks coming from independent cohorts [43] to efficiently distinguish the preserved from the non-preserved modules. Finally, hub genes within the candidate modules are chosen by considering both connectivity-based and trait-based significance measures. Module membership for a specific gene is computed as a Pearson correlation between its expression profile and the specific module eigengene (Supplementary Methods) and signifies the connectivity-based importance of the gene within a module of interest. In this study, we used the phenotype status of the samples in both the cohorts along with DL_CO_ and FVC lung function traits from the GSE47460 training cohort to calculate trait-based gene significance (see Supplementary Methods for additional details).

### 4.8. Immunohistochemistry

De-identified lung tissue specimens were collected from the distal areas of explanted lungs using research protocol approved by the Cincinnati Children’s Hospital Medical Center institutional review board (IRB # 2015-1347). The lung tissues were fixed in formalin and embedded with paraffin to prepare 6-micron-thick lung tissue sections from both IPF and age-matched healthy controls (*n* = 6–7/group). The lung sections were immunostained with anti-CRABP2 (Sigma, St. Louis, MO, USA) as a primary antibody (1:200 dilution). We used goat anti-rabbit Ig as an isotype control antibody and observed no detectable immunostaining (data not shown). Hematoxylin counterstain was used to counter stain nuclei in color blue. All images were collected using a Keyence BZ-X800 microscope (Itasca, IL, USA) at high magnification (×60). BZ-X image analysis software was used to quantify brown staining areas in the total lung area of five representative images collected for each lung slide and expressed as the percentage of CRABP2-positive area in the total lung area of an image.

## Figures and Tables

**Table 1:** List of selected novel candidate genes found to be associated with DL_CO_ and FVC/FEV lung function traits (from Open Targets Genetics portal and GWAS Catalog). Also reported are the correlations computed between the expression levels of the candidate genes and the lung function trait measurements from GSE47460 and GSE150910 [24].

Gene	GSE47460				GSE150910			
	DL_CO_	*p*-Value	FVC	*p*-Value	DL_CO_	*p*-value	FVC	*p*-Value
Upregulated in IPF								
***IGF1***	−0.6	7.42 × 10^−26^	−0.54	5.68 × 10^−20^	−0.55	6.51 × 10^−16^	−0.5	4.95 × 10^−13^
***LTBP1***	−0.61	7.23 × 10^−27^	−0.55	8.62 × 10^−21^	−0.33	4.91 × 10^−6^	−0.31	2.32 × 10^−5^
***SULF1***	−0.61	7.23 × 10^−27^	−0.55	8.62 × 10^−21^	−0.54	1.34 × 10^−15^	−0.54	4.64 × 10^−15^
***COL15A1***	−0.59	7.59 × 10^−25^	−0.55	8.62 × 10^−21^	−0.33	9.12 × 10^−6^	−0.18	0.0249
***SERPINF1***	−0.65	2.85 × 10^−31^	−0.59	2.91 × 10^−24^	−0.57	2.46 × 10^−17^	−0.47	1.60 × 10^−11^
***PDIA4***	−0.65	2.85 × 10^−31^	−0.57	1.77 × 10^−22^	−0.49	1.57 × 10^−12^	−0.46	4.54 × 10^−11^
***COL10A1***	−0.61	7.23 × 10^−27^	−0.57	1.77 × 10^−22^	−0.49	1.15 × 10^−12^	−0.58	1.41 × 10^−17^
***COL14A1***	−0.64	4.43 × 10^−30^	−0.54	5.68 × 10^−20^	−0.55	4.21 × 10^−16^	−0.5	4.72 × 10^−13^
***COL18A1***	−0.61	7.23 × 10^−27^	−0.58	2.27 × 10^−23^	−0.45	2.46 × 10^−10^	−0.4	3.38 × 10^−8^
***SCRG1***	−0.62	6.88 × 10^−28^	−0.56	1.28 × 10^−21^	−0.41	6.28 × 10^−9^	−0.35	1.67 × 10^−6^
***GPX8***	−0.59	7.59 × 10^−25^	−0.56	1.28 × 10^−21^	−0.3	3.65 × 10^−5^	−0.31	2.97 × 10^−5^
***COL3A1***	−0.58	6.83 × 10^−24^	−0.55	8.62 × 10^−21^	−0.48	3.25 × 10^−12^	−0.51	1.75 × 10^−13^
***STEAP2***	−0.59	7.59 × 10^−25^	−0.52	2.07 × 10^−18^	−0.68	7.19 × 10^−27^	−0.63	1.24 × 10^−21^
***STEAP1***	−0.65	2.85 × 10^−31^	−0.6	3.15 × 10^−25^	−0.64	4.73 × 10^−23^	−0.59	2.23 × 10^−18^
***TTC39C***	−0.66	2.13 × 10^−32^	−0.57	1.77 × 10^−22^	−0.47	2.14 × 10^−11^	−0.41	1.42 × 10^−8^
***ITGA7***	−0.59	7.59 × 10^−25^	−0.51	1.17 × 10^−17^	−0.46	3.61 × 10^−11^	−0.41	1.67 × 10^−8^
***ZNF385D***	−0.61	7.23 × 10^−27^	−0.53	3.50 × 10^−19^	−0.53	1.51 × 10^−14^	−0.44	7.71 × 10^−10^
***DCLK1***	−0.61	7.23 × 10^−27^	−0.53	3.50 × 10^−19^	−0.5	4.62 × 10^−13^	−0.48	6.39 × 10^−12^
***CFI***	−0.61	7.23 × 10^−27^	−0.51	1.17 × 10^−17^	−0.62	2.30 × 10^−21^	−0.53	2.43 × 10^−14^
**Downregulated in IPF**								
***ADRB2***	0.67	1.24 × 10^−33^	0.6	3.15 × 10^−25^	0.69	5.22 × 10^−28^	0.63	5.79 × 10^−22^
***WNT7A***	0.64	4.43 × 10^−30^	0.6	3.15 × 10^−25^	0.59	8.33 × 10^−19^	0.56	1.33 × 10^−16^
***AFF3***	0.61	7.23 × 10^−27^	0.52	2.07 × 10^−18^	0.65	6.70 × 10^−24^	0.51	1.10 ×10^−13^
***AGER***	0.7	1.50 × 10^−37^	0.64	2.81 × 10^−29^	0.7	1.36 × 10^−28^	0.66	1.80 × 10^−24^
***MATN3***	0.61	7.23 × 10^−27^	0.52	2.07 × 10^−18^	0.25	0.0007	0.2	0.0105
***NINJ2***	0.65	2.85 × 10^−31^	0.6	3.15 × 10^−25^	0.67	2.67 × 10^−25^	0.59	8.19 × 10^−19^
***FRY***	0.66	2.13 × 10^−32^	0.58	2.27 × 10^−23^	0.61	1.42 × 10^−20^	0.55	3.61 × 10^−16^
***ARHGAP31***	0.64	4.43 × 10^−30^	0.55	8.62 × 10^−21^	0.57	1.24 × 10^−17^	0.5	1.03 × 10^−12^
***ARHGEF26***	0.61	7.23 × 10^−27^	0.55	8.62 × 10^−21^	0.63	8.08 × 10^−22^	0.53	1.25 × 10^−14^
***ANKS1A***	0.6	7.42 × 10^−26^	0.5	6.32 × 10^−17^	0.2	0.0104	0.21	0.0061
***CCBE1***	0.62	6.88 × 10^−28^	0.56	1.28 × 10^−21^	0.59	1.75 × 10^−18^	0.44	5.15 × 10^−10^
***NCKAP5***	0.65	2.85 × 10^−31^	0.62	3.05 × 10^−27^	0.63	7.77 × 10^−22^	0.61	4.24 × 10^−20^
***EPB41L5***	0.61	7.23 × 10^−27^	0.55	8.62 × 10^−21^	0.66	3.84 × 10^−25^	0.62	1.89 × 10^−20^
***ANXA3***	0.66	2.13 × 10^−32^	0.59	2.91 × 10^−24^	0.65	5.04 × 10^−24^	0.61	3.39 × 10^−20^
***EMP2***	0.68	7.32 × 10^−35^	0.63	3.15 × 10^−28^	0.3	4.57 × 10^−5^	0.21	0.0056
***RTKN2***	0.66	2.13 × 10^−32^	0.61	3.18 × 10^−26^	0.7	8.68 × 10^−29^	0.66	1.72 × 10^−24^
***SPRYD7***	0.66	2.13 × 10^−32^	0.61	3.18 × 10^−26^	0.52	3.90 × 10^−14^	0.47	2.00 × 10^−11^
***SLC44A2***	0.58	6.83 × 10^−24^	0.53	3.50 × 10^−19^	0.56	7.94 × 10^−17^	0.55	5.85 × 10^−16^
***KCNMB4***	0.62	6.88 × 10^−28^	0.53	3.50 × 10^−19^	0.53	5.99 × 10^−15^	0.48	8.93 × 10^−12^
***FAM167A***	0.67	1.24 × 10^−33^	0.59	2.91 × 10^−24^	0.6	1.84 × 10^−19^	0.49	1.45 × 10^−12^
***OLFML2A***	0.68	7.32 × 10^−35^	0.61	3.18 × 10^−26^	0.67	4.13 × 10^−26^	0.67	3.79 × 10^−25^
***ECHDC3***	0.57	5.91 × 10^−23^	0.54	5.68 × 10^−20^	0.2	0.0086	0.092	0.2773
***SEMA3B***	0.66	2.13 × 10^−32^	0.62	3.05 × 10^−27^	0.48	5.53 × 10^−12^	0.52	5.33 × 10^−14^
***LAMA3***	0.64	4.42 × 10^−30^	0.57	1.77 × 10^−22^	0.62	1.62 × 10^−21^	0.6	1.34 × 10^−19^
***PCYOX1***	0.65	2.85 × 10^−31^	0.57	1.77 × 10^−22^	0.17	0.03216	0.17	0.03719
***RNF144B***	0.6	7.42 × 10^−26^	0.54	5.68 × 10^−20^	0.5	5.28 × 10^−13^	0.42	3.36 × 10^−9^
***HYAL1***	0.58	6.83 × 10^−24^	0.53	3.50 × 10^−19^	0.54	9.30 × 10^−16^	0.47	1.55 × 10^−11^
***CDH13***	0.62	6.88 × 10^−28^	0.59	2.91 × 10^−24^	0.44	6.94 × 10^−10^	0.41	1.45 × 10^−8^
***CTNND2***	0.71	4.57 × 10^−39^	0.64	2.81 × 10^−29^	0.63	3.33 × 10^−22^	0.57	6.84 × 10^−17^
***DPP6***	0.69	3.50 × 10^−36^	0.62	3.05 × 10^−27^	0.65	3.78 × 10^−24^	0.61	7.69 × 10^−20^
***GRIA1***	0.67	1.24 × 10^−33^	0.62	3.05 × 10^−27^	0.67	6.26 × 10^−26^	0.65	1.80 × 10^−23^
***DENND3***	0.64	4.43 × 10^−30^	0.57	1.77 × 10^−22^	0.46	4.49 × 10^−11^	0.38	1.25 × 10^−7^

**Table 2:** Novel intramodular hubs which are differentially expressed across different IPF stages (GSE124685 [25]). Estimates of log2 fold changes (≥0.6 log FC or ≤−0.6 log FC; adjusted *p*-value < 0.05) in each stage are listed for each candidate gene (see Supplementary Table S7 for additional details).

Hub Gene	Name	IPF1—Early logFC	IPF2—Progressive logFC	IPF3—Advanced logFC
Brown Module				
*CDH3*	Cadherin 3	2.16	2.53	2.59
*CFI*	Complement factor I	1.01	0.7	0.95
*CHRDL2*	Chordin like 2	1.03	2.04	1.8
*COL10A1*	Collagen type X alpha 1 chain	2.78	2.84	2.91
*CRABP2*	Cellular retinoic acid binding protein 2	2.45	3	3.25
*DCLK1*	Doublecortin-like kinase 1	0.86	0.94	1.04
*DOK5*	Docking protein 5	1.16	1.37	1.23
*FNDC4*	Fibronectin type III domain containing 4	1.04	1.19	1.29
*GPX8*	Glutathione peroxidase 8 (putative)	1.08	0.98	1.16
*SCRG1*	Stimulator of chondrogenesis 1	1.23	1.29	1.48
*SPRR1A*	Small proline rich protein 1A	2.51	3.19	3.66
*STEAP1*	STEAP family member 1	1.3	1.57	1.74
*STEAP2*	STEAP2 metalloreductase	1.03	1.35	1.34
*TDO2*	Tryptophan 2,3-dioxygenase	2.34	2.92	2.8
**Blue Module**				
*AATK*	Apoptosis associated tyrosine kinase	−0.94	−0.96	−0.99
*AFF3*	AF4/FMR2 family member 3	−1.22	−1.43	−1.44
*ARHGEF26*	Rho guanine nucleotide exchange factor 26	−0.91	−1.14	−1.27
*BTNL9*	Butyrophilin like 9	−3.39	−4	−3.69
*C1orf115*	Chromosome 1 open reading frame 115	−0.85	−1.14	−1.26
*CDH13*	Cadherin 13	−0.69	−0.84	−0.88
*CRTAC1*	Cartilage acidic protein 1	−1.57	−2	−2.33
*DENND3*	DENN domain containing 3	−1.17	−1.16	−1.33
*EMP2*	Epithelial membrane protein 2	−0.74	−1.4	−1.53
*EPB41L5*	Erythrocyte membrane protein band 4.1 like 5	−0.81	−1.04	−1.1
*GALNT18*	Polypeptide N-acetylgalactosaminyltransferase 18	−1.33	−1.6	−1.71
*GRIA1*	Glutamate ionotropic receptor AMPA type subunit 1	−0.71	−1.1	−1.09
*HPCAL1*	Hippocalcin like 1	−0.86	−1.34	−1.71
*ITLN2*	Intelectin 2	−2.29	−3.52	−4.18
*KANK3*	KN motif and ankyrin repeat domains 3	−0.92	−1.08	−1.16
*KCNMB4*	Potassium calcium-activated channel subfamily M regulatory beta subunit 4	−1.02	−1.36	−1.41
*MATN3*	Matrilin 3	−0.92	−1.4	−1.61
*MYRF*	Myelin regulatory factor	−1.43	−1.59	−2.13
*NDRG4*	NDRG family member 4	−1.59	−2.24	−2.61
*NPR1*	Natriuretic peptide receptor 1	−0.79	−1.22	−1.27
*OLFML2A*	Olfactomedin like 2A	−0.85	−0.94	−0.9
*PAPSS2*	3′-phosphoadenosine 5′-phosphosulfate synthase 2	−0.66	−1.01	−1.3
*PLLP*	Plasmolipin	−0.86	−1.49	−1.7
*RNF144B*	Ring finger protein 144B	−0.65	−0.91	−1.17
*RS1*	Retinoschisin 1	−0.71	−1.61	−1.79
*SERTM1*	Serine rich and transmembrane domain containing 1	−1.11	−1.8	−2.02
*STARD8*	StAR related lipid transfer domain containing 8	−0.95	−1.08	−1.29
*STXBP6*	Syntaxin binding protein 6	−0.76	−1.71	−2.17
*VIPR1*	Vasoactive intestinal peptide receptor 1	−1.51	−2.41	−2.72
*VSIG10*	V-set and immunoglobulin domain containing 10	−0.75	−1.09	−0.96

**Table 3:** List of novel candidate genes capable of distinguishing fibrotic samples from healthy controls in the two training cohorts and two independent validation cohorts (GSE150910 and GSE134692).

Symbol	Gene ID	Description
Brown Module		
***CHEK2***	11200	Checkpoint kinase 2
***CRABP2***	1382	Cellular retinoic acid binding protein 2
***TSHZ2***	128553	Teashirt zinc finger homeobox 2
***COL7A1***	1294	Collagen type VII alpha 1 chain
***SEC24D***	9871	SEC24 homolog D, COPII coat complex component
***REEP2***	51308	Receptor accessory protein 2
***COL10A1***	1300	Collagen type X alpha 1 chain
***TTC39C***	125488	Tetratricopeptide repeat domain 39C
***STEAP1***	26872	STEAP family member 1
***EFNA4***	1945	Ephrin A4
***CLMP***	79827	CXADR-like membrane protein
***CDH3***	1001	Cadherin 3
***NPM3***	10360	Nucleophosmin/nucleoplasmin 3
***VWCE***	220001	von Willebrand factor C and EGF domains
***PLEKHA4***	57664	Pleckstrin homology domain containing A4
***CFI***	3426	Complement factor I
***TDO2***	6999	Tryptophan 2,3-dioxygenase
***TMEM229A***	730130	Transmembrane protein 229A
**Blue Module**		
***MATN3***	4148	Matrilin 3
***FRY***	10129	FRY microtubule binding protein
***CTNND2***	1501	Catenin delta 2
***RADIL***	55698	Rap associating with DIL domain
***ECHDC3***	79746	Enoyl-CoA hydratase domain containing 3
***KANK3***	256949	KN motif and ankyrin repeat domains 3
***SPRING1***	79794	SREBF pathway regulator in golgi 1
***ANKS1A***	23294	Ankyrin repeat and sterile alpha motif domain containing 1A
***SLC44A2***	57153	Solute carrier family 44 member 2
***TNS3***	64759	Tensin 3
***ST6GALNAC5***	81849	ST6 N-acetylgalactosaminide alpha-2,6-sialyltransferase 5
***C5orf38***	153571	Chromosome 5 open reading frame 38
***AFF3***	3899	AF4/FMR2 family member 3
***RNF182***	221687	Ring finger protein 182
***CRTAC1***	55118	Cartilage acidic protein 1
***PLLP***	51090	Plasmolipin
***NINJ2***	4815	Ninjurin 2
***KCNMB4***	27345	Potassium calcium-activated channel subfamily M regulatory beta subunit 4
***VSIG10***	54621	V-set and immunoglobulin domain containing 10
***PDZD2***	23037	PDZ domain containing 2
***BTNL9***	153579	Butyrophilin like 9
***VIPR1***	7433	Vasoactive intestinal peptide receptor 1
***DENND3***	22898	DENN domain containing 3
***FAM189A1***	23359	Family with sequence similarity 189 member A1

**Table 4:** Top 20 ranked novel hubs ranked based on their functional similarities to known GWA IPF genes. Upregulated and downregulated hubs are ranked separately.

Rank	Upregulated Hub Genes	Description	Downregulated Hub Genes	Description
**1**	*CHEK2*	Checkpoint kinase 2	*CTNND2*	Catenin delta 2
**2**	*CDH3*	Cadherin 3	*CDH13*	Cadherin 13
**3**	*COL7A1*	Collagen type VII alpha 1 chain	*SELENBP1*	Selenium binding protein 1
**4**	*CFI*	Complement factor I	*ARHGAP31*	Rho GTPase activating protein 31
**5**	*KCND3*	Potassium voltage-gated channel subfamily D member 3	*CAVIN2*	Caveolae associated protein 2
**6**	*CRABP2*	Cellular retinoic acid binding protein 2	*DENND3*	DENN domain containing 3
**7**	*ZNF469*	Zinc finger protein 469	*SLC1A1*	Solute carrier family 1 member 1
**8**	*STEAP2*	STEAP2 metalloreductase	*EMP2*	Epithelial membrane protein 2
**9**	*TDO2*	Tryptophan 2,3-dioxygenase	*PAPSS2*	3′-phosphoadenosine 5′-phosphosulfate synthase 2
**10**	*SEC24D*	SEC24 homolog D, COPII coat complex component	*SLC44A2*	Solute carrier family 44 member 2
**11**	*CLMP*	CXADR like membrane protein	*N4BP1*	NEDD4 binding protein 1
**12**	*DCLK1*	Doublecortin like kinase 1	*GPM6A*	Glycoprotein M6A
**13**	*MAGED4B*	MAGE family member D4B	*NINJ2*	Ninjurin 2
**14**	*PDIA4*	Protein disulfide isomerase family A member 4	*RRAS*	RAS related
**15**	*ITGA7*	Integrin subunit alpha 7	*HPCAL1*	Hippocalcin like 1
**16**	*NPM3*	Nucleophosmin/nucleoplasmin 3	*AFF3*	AF4/FMR2 family member 3
**17**	*COL10A1*	Collagen type X alpha 1 chain	*NDRG4*	NDRG family member 4
**18**	*GPX8*	Glutathione peroxidase 8 (putative)	*VIPR1*	Vasoactive intestinal peptide receptor 1
**19**	*EFNA4*	Ephrin A4	*MYRF*	Myelin regulatory factor
**20**	*DOK5*	Docking protein 5	*RS1*	Retinoschisin 1

**Table 5:** List of GEO datasets used in the WGCNA consensus analysis (including the validation cohorts).

GEO Accession ID	# IPF Cases	# Controls	Reference
GSE47460	160	108	LGRC
GSE53845	40	8	[32]
GSE134692	46	26	[33]
GSE150910	103	103	[24]

## Data Availability

All data generated or analyzed during this study are included in this article and its supplemental information files.

## Supplementary Materials

The following supporting information can be downloaded at: https://www.mdpi.com/article/10.3390/ijms23105447/s1.
