# Supplementary material for: Consensus Gene Co-Expression Network Analysis Identifies Novel Genes Associated with Severity of Fibrotic Lung Disease
Source: Int J Mol Sci. 2022 May 13;23(10):5447. doi: 10.3390/ijms23105447 (PMC9141193; doi:10.3390/ijms23105447)
Supplement: Supplementary file 1 [file ijms-23-05447-s001.zip › ijms-1702128-supplementary Figures.pdf]

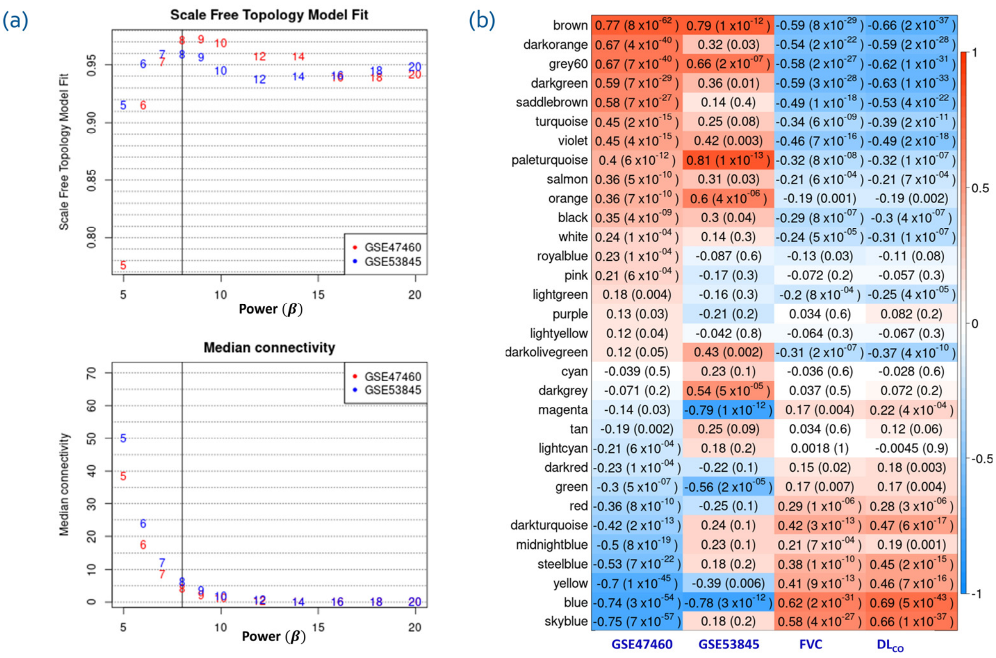

Figure S1. (A) Scatter plots of scale free criterion model fit (top) and median node connectivity (bottom) from the input gene co-expression networks as a function of the power adjacency function parameter (β) **(B)** Correlation heatmap of all 32 consensus modules with the phenotypic traits from both training datasets.

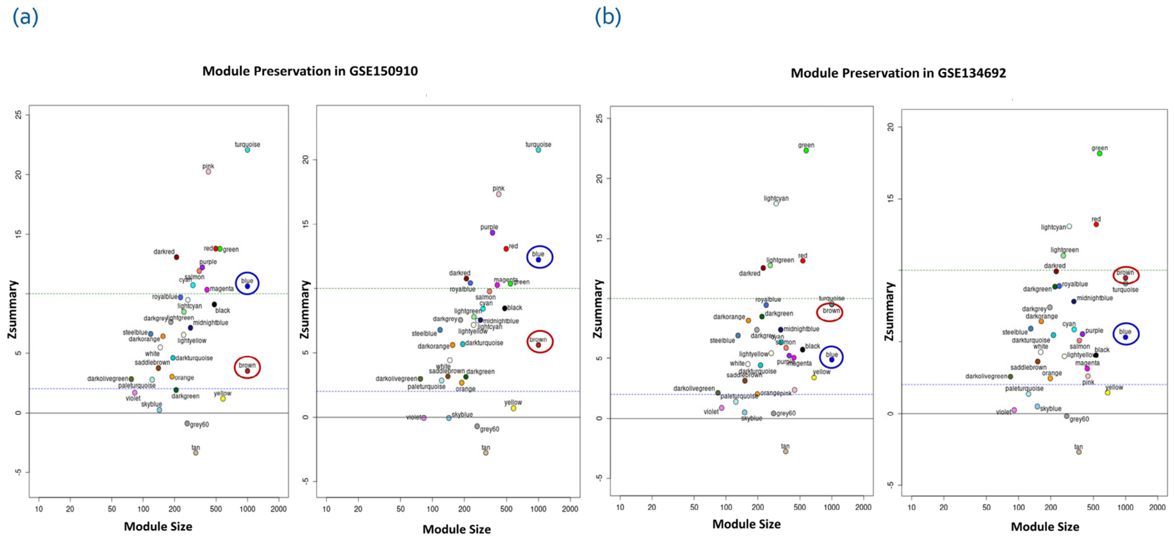

Figure S2. Module preservation scatter plots of consensus modules in two independent cohorts (GSE150910 – Panel (**a**) and GSE134692 – Panel (**b**)). Two scatter plots in each panel correspond to separate module preservation analysis experiments with either of the two training cohorts as "reference" studies. Each point in the scatter plot is the $Z_{summary}$ score associated with a given consensus module. The module sizes are along the x-axis in each plot.

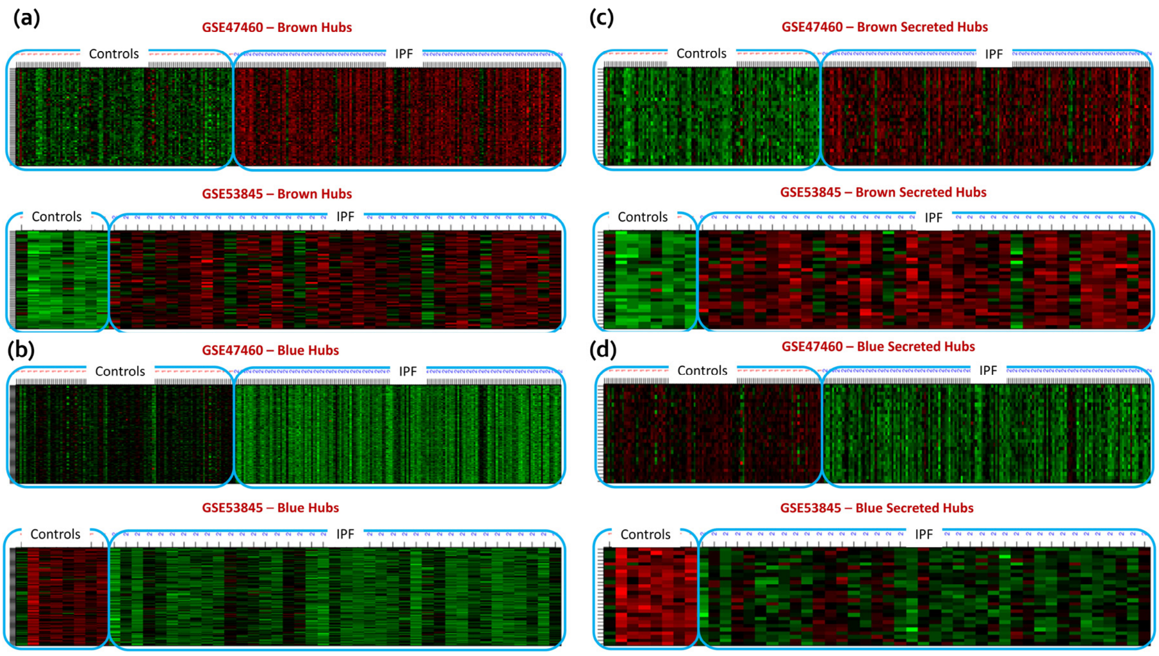

Figure S3. Panel of heatmaps of both the training cohorts (LGRC and GSE53845) using the complete set of intramodular hubs (Panels (**a**) and (**b**)) or the secreted hubs subset (Panels (**c**) and (**d**)).

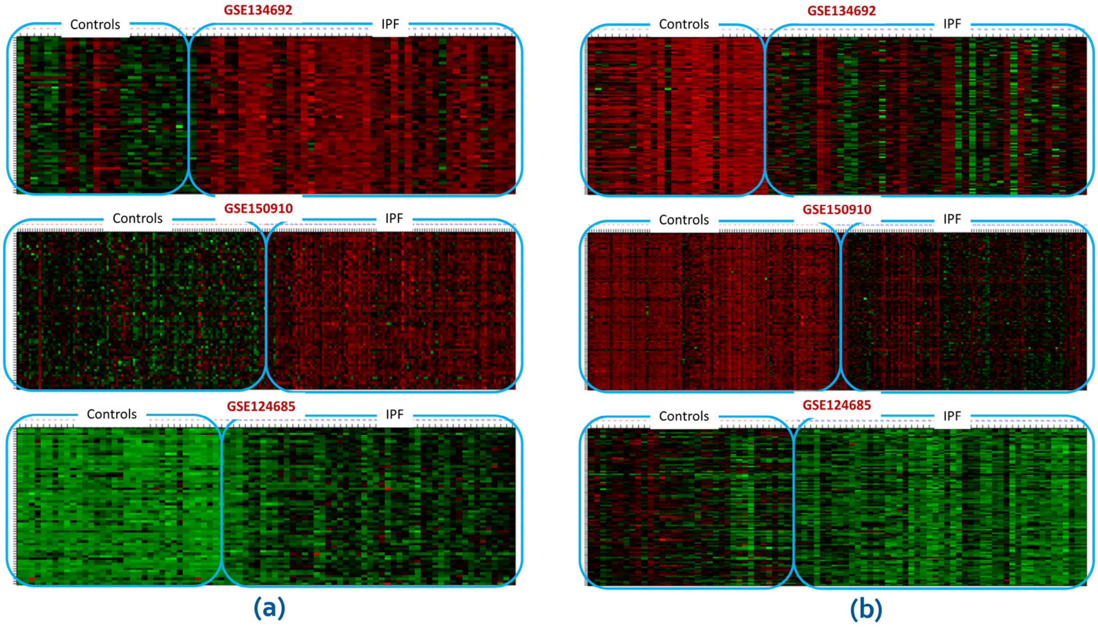

Figure S4. Panel of expression heatmaps from the validation cohorts (GSE134692, GSE150910 and GSE124685)) using intramodular hubs genes from the brown (Panel (**a**)) and blue (Panel (**b**)) candidate modules.

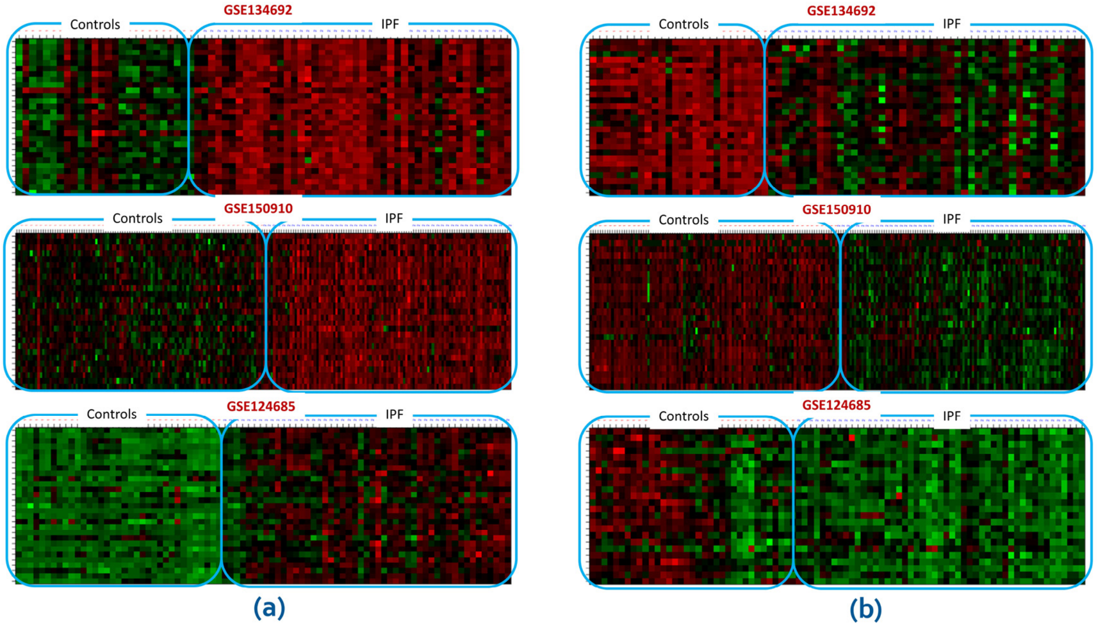

Figure S5. Panel of heatmaps from the validation cohorts (GSE134692, GSE150910 and GSE124685)) using secreted intramodular hubs genes from the brown (Panel (**a**)) and blue (Panel (**b**)) candidate modules. The list of secreted proteins is obtained from the Human Protein Atlas.

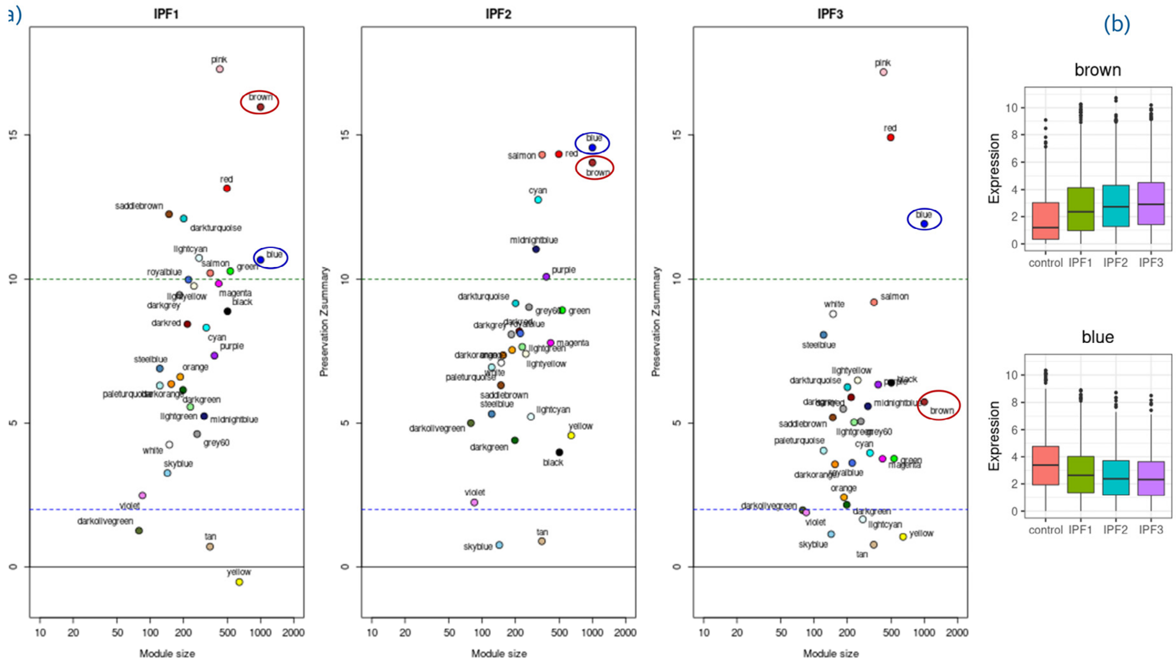

Figure S6. Panel of module preservation scatter plots of consensus modules (Panel (**a**)) in three different stages of IPF (Early or IPF-1, Progressive or IPF-2, and advanced or IPF-3; GSE124685). Each point in the scatter plot is the $Z_{summary}$ score associated with a given consensus module. Training cohort GSE47460 is used as the "reference" study in all three of these experiments. Box plots of module expression levels in the three IPF subtypes are also included (Panel (**b**)).

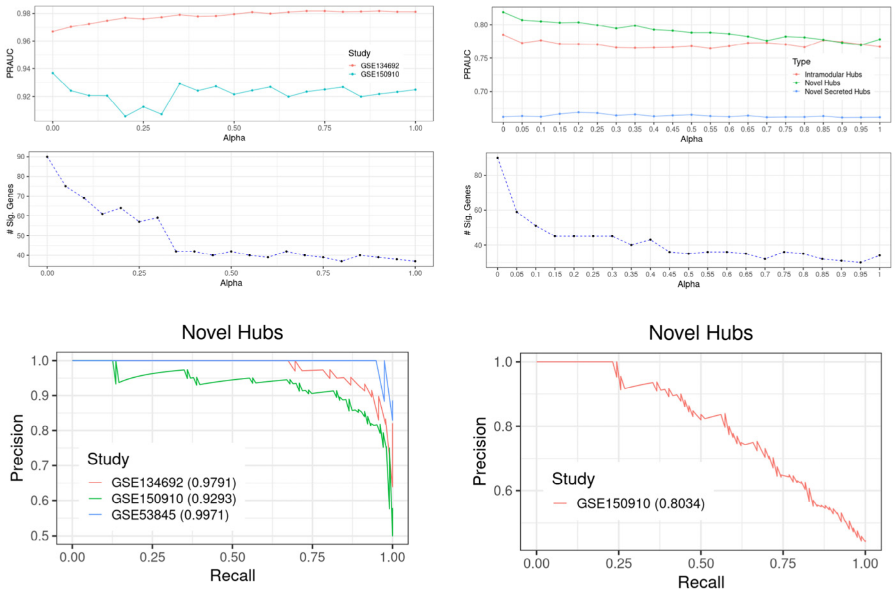

(a) **(b)**

Figure S7. Panel plots associated regularized logistic regression models for distinguishing IPF samples from other interstitial lung disorders. Panel (**a**) contains the results from our experiments in the two independent validation studies (GSE134692 and GSE150910) for distinguishing IPF samples from healthy controls. The scatter plots (on the top) were used to choose the optimal regularization penalty parameter (α) for training the regression models. Precision-Recall Curve (bottom) associated with the best-performing model is also included. Similarly, Panel (**b**) contains the results from our experiments to classify the IPF patients along with chronic hypersensitive pneumonitis (CHP) samples.
