# Supplementary material for: Consensus Gene Co-Expression Network Analysis Identifies Novel Genes Associated with Severity of Fibrotic Lung Disease
Source: Int J Mol Sci. 2022 May 13;23(10):5447. doi: 10.3390/ijms23105447 (PMC9141193; doi:10.3390/ijms23105447)
Supplement: Supplementary file 1 [file ijms-23-05447-s001.zip › Supplementary Methods.pdf]

SUPPLEMENTARY METHODS

Weighted gene co-expression analysis

Weighted gene co-expression network analysis (WGCNA) frameworks helps in identifying clusters of co-expressed genes [1] based on pairwise-correlations between gene expression profiles across all samples (eq. 1). For each expression dataset used, a signed weighted adjacency matrix is computed based on co-expression similarities between the genes (eq. 2) as shown below:

$$s_{ij} = \frac{1+cor(i,j)}{2} \quad (1)$$

$$a_{ij} = power(s_{ij}, \beta) = |s_{ij}|^{\beta} \quad (2)$$

where $cor(i, j)$ is the Pearson correlation coefficient between the expression profiles of a pair of genes i & j:

$$cor(i,j) = \frac{\sum_{k=1}^{n}(i_k-\bar{i})(j_k-\bar{j})}{\sqrt{\sum_{k=1}^{n}(i_k-\bar{i})^2}\sqrt{\sum_{k=1}^{n}(j_k-\bar{j})^2}} \quad (3)$$

where $\vec{i}$ and $\vec{j} \in R^n$ are the respective expression vectors ($n =$ number of samples). The parameter β in the power adjacency function (eq. 2) is chosen based on the scale-free topology criterion [1]. These adjacencies are further used to compute topological overlaps between two genes which reflect their level of interconnectedness in the co-expression network (eq. 4).

$$TOM_{ij}(A) = \frac{\sum_{k \neq i,j} a_{ik}a_{kj} + a_{ij}}{min(\sum_{k \neq i} a_{ik}, \sum_{k \neq j} a_{jk}) + 1 - a_{ij}} \quad (4)$$

Average linkage hierarchical clustering is then performed on TOM-based dissimilarities to detect modules of highly correlated genes across the samples.

Consensus network analysis

WGCNA consensus module analysis can be used to find highly connected gene modules from multiple transcriptomic studies [2]. It involves constructing co-expression networks for each dataset and then identifying consensus modules among them, consisting of genes closely connected in all networks. These modules are hypothesized to represent pathways or biological processes shared among the different studies under analysis. Topological similarity matrices are constructed for each dataset and then scaled to bring their distributions closer. In this study, we considered the consensus network to be the component-wise minimum of the individual networks i.e., the topological similarity matrices from each of the two datasets (eq. 5 and eq. 6). These individual TOM matrices were scaled such that their 95th percentiles are the same.

$$\boldsymbol{Consensus}\{\boldsymbol{TOM}(\boldsymbol{A}^{GSE47460}),\ \boldsymbol{TOM}(\boldsymbol{A}^{GSE53845})\} = \boldsymbol{Min}_{ij}\{\boldsymbol{TOM}(\boldsymbol{A}^{GSE47460}),\ \boldsymbol{TOM}(\boldsymbol{A}^{GSE53845})\} \quad (5)$$

$$\boldsymbol{Dissim}(\boldsymbol{Consensus}\{\boldsymbol{TOM}(\boldsymbol{A}^{GSE47460}),\ \boldsymbol{TOM}(\boldsymbol{A}^{GSE53845})\}) = \mathbf{1} - \boldsymbol{Consensus}\{\boldsymbol{TOM}(\boldsymbol{A}^{GSE47460}),\ \boldsymbol{TOM}(\boldsymbol{A}^{GSE53845})\} \quad (6)$$

This consensus similarity matrix is used as input to average linkage hierarchical clustering to obtain co-expressed gene modules. WGCNA employs an adaptive branch pruning of hierarchical clustering dendrograms [3].

Prioritizing consensus modules

WGCNA identified modules can be ranked and prioritized by relating them with external sample information such as clinical traits and phenotype status. A representative summarizing the module expression profile is chosen and correlated with the traits of choice. In general, the first principal component, referred to as module eigengene, is used as the representative of the entire co-

expression module. Disease-related consensus modules are selected based on the eigengene significance $MES^{(q)}$ (eq. 7) across the individual datasets.

$$\boldsymbol{MES^{(q)}} = \boldsymbol{Cor}\left(\boldsymbol{E^{(q)}}, \boldsymbol{T}\right) \quad (7)$$

$MES^{(q)}$ is simply defined as correlation between the module eigengene $E^{(q)}$ of the specific module and disease-status denoted by the vector T. Candidate modules are determined based on the strength and significance of these module correlations.

Module preservation analysis

Module preservation analysis in WGCNA [4] can be used to obtain the preservation status of prioritized candidate modules in independent test cohorts. It considers the identified module memberships as supervised labels and computes different statistical metrics associated with conservation status of the modules. These include both density-based and connectivity-based metrics. Then, individual Z statistics are computed for these metrics using permutation tests where the module assignments are randomly permuted. These Z statistics are aggregated into a composite score ($Z_{summary}$) which is then used to assess the preservation status for each module. These composite preservation statistics have been shown to efficiently distinguish the preserved from the non-preserved gene modules. Empirical evidence from simulation studies [4] have shown that modules with $Z_{summary} > 10$ are strongly preserved while those with $2 < Z_{summary} < 10$ can be considered to be moderately preserved. Finally, if $Z_{summary} < 2$, then there is no statistical evidence that the module is preserved.

Identifying intramodular hubs

Intramodular hubs in disease-related candidate modules are often shown to be of high clinical importance. They are generally chosen by considering connectivity-based and/or trait-based significance measures. Module membership score for a specific gene is computed as Pearson correlation between its expression profile and the specific module eigengene (eq. 8) and signifies the connectivity-based importance of the gene within a module of interest

$$\boldsymbol{kME_i^{(q)} = Cor(\overrightarrow{x_i}, E^q)} \quad (8)$$

where $\overrightarrow{\boldsymbol{x_i}}$ is the expression profile of gene i and E^q is the eigengene of module **q**. Similarly, a trait-based gene significance is measured as the correlation between the expression profile and the clinical trait (eq. 9).

$$\boldsymbol{GS_i^T = Cor(\overrightarrow{x_i}, T)} \quad (9)$$

where $\overrightarrow{\boldsymbol{x_i}}$ again is the expression profile of the ith gene and T is clinical trait [5]. In this study we used the phenotype status of samples in both the cohorts along with DLCO and FVC lung function traits from GSE47460 in calculating trait-based gene significances. In case of consensus modules, we considered the weighted average kME^i (eq. 8) for each gene i in the corresponding module, across the input data sets. The weight from each data set is proportional to number of samples in the dataset [6]. Finally, we defined a $HubScore_i$ for each gene as the $Harmonic\ Mean$ of both connectivity-based and trait-based significances defined above (eq. 10).

$$\boldsymbol{HubScore_i = HarmonicMean(kME_i^{(q)}, GS_i^{Phenotype}, GS_i^{DLCO}, GS_i^{FVC})} \quad (10)$$

Hence, hub genes identified using the above score are hypothesized to be network hubs, strongly associated with the phenotypic traits of choice.

Consensus gene modules in IPF

Normalized gene expression profiles from whole lung tissues from two training cohorts, GSE47460 and GSE53845 [7], were extracted from the NCBI GEO [7] repository. Prior to applying the preprocessing steps, sample characteristics and traits were retrieved from the raw expression matrix files. Additionally, the LTRC dataset (GSE47460) was further filtered to retain only the IPF samples (n=160) and controls (n=108). Expression profiles of 15,180 genes found in both the datasets were used as inputs to the WGCNA consensus analysis. Signed pairwise Pearson correlations (eq. 3) were computed individually in each dataset and converted into weighted gene-gene adjacencies using the power adjacency function (eq. 2). The estimated value of the single parameter β was chosen based on the scale-free topology criterion (Supplementary Figure S1a). This weighted adjacency matrix now represents the gene co-expression network with the signed Pearson correlations raised to the chosen power β = 8, as edge weights. Using these co-expression networks, topological overlap-based similarities (eq. 4) are calculated for each pair of genes reflecting their relative interconnectedness in each network. Further, these topological similarities are quantile transformed before combining them, forming the consensus network. Finally, hierarchical clustering is performed on the consensus dissimilarities $(1 - consensus\_sim)$ with the dynamic tree cut method [3] used to identify gene modules. The cluster sensitivity parameter (*deepSplit*) was set to the default value of 2 to achieve balanced clusters with respect to the gene counts. Subsequently, from the set of 15,180 commonly found genes, we identified 32 consensus gene modules. Genes within these modules are hypothesized to be co-expressed in samples from both the studies. Sizes of these consensus modules have been observed to be anywhere in between 100 – 2000 genes.

Then, module preservation analysis was applied on two independent test cohorts, GSE134692 [8] and GSE150910 [9] and the summarized preservation statistics ($Z_{summary}$) are computed for each consensus module by aggregating both density-based and connectivity-based measures. Specifically, connectivity-based preservation statistics quantify how close the connectivity of genes from a given module is between a designated reference network and a test network. Since the modules in this study come from two different training networks, we repeated the analysis using two different reference studies (Supplementary Figure S2). Before the aggregation step, Z statistics are computed for each metric using 200 random permutations of each individual test network. Finally, composite $Z_{summary}$ scores are used to identify candidate consensus modules that are not only associated with the phenotype status and lung function traits but also conserved across different studies (Figure S1). All the pre-processing and analysis steps described above were implemented using the WGCNA R package [10].

Regularized logistic regression models with elastic net penalty

In this study, we filtered novel candidate hub genes from the consensus modules by designing and training several regularized logistic regression models with the regularization parameter λ. In all these models, the gene expression levels are used as continuous predictor variables to predict the phenotype status (outcomes). Elastic net penalty linearly combines and controls both L1 and L2 regularization penalties using a mixing parameter α. It bridges the gap between lasso regression ($\alpha = 1$) and ridge regression ($\alpha = 0$) models. In all our experiments, we employed a grid search over different values of the α parameter ranging between 0 and 1, over increments of 0.05. For each α value, we further tested different values of the λ parameter, ranging between 0.001 and 100

and identified the best value using 3-fold cross validation on the training data. All these experiments were implemented using the *cv.glmnet* method in the glmnet R package [11]. Finally, we evaluated these models (associated with each α value) on independent test cohorts or partitions using different evaluation metrics. Randomized trials were conducted to assess the significance of these evaluation metrics by randomly choosing the gene predictors and computing false discovery rates (FDR) of the observed scores. To choose the best-performing models (i.e., the best α value), we not only considered the test evaluation metrics and their significance but also the number of significant genes/features used as predictors. The idea was to identify "lean" models that also performed well in our evaluations to avoid any potential overfitting.

Our first set of experiments were designed to identify candidate genes capable of classifying IPF samples from healthy controls. We trained binary logistic regression models on 268 LGRC samples (160 IPF samples and 108 controls) and evaluated them on two independent test cohorts (GSE134692 -> 46 IPF, 26 controls and GSE150910 -> 103 IPF, 103 controls). For evaluation, we constructed precision-recall (PR) curves and computed the area under the curve (AUC) scores. We further assessed the observed PRAUC scores using 10,000 times randomized trials for each trained model.

The next set of models we trained were to identify potential biomarkers that can be used to distinguish IPF from chronic hypersensitive pneumonitis (CHP). We have used 160 IPF and 30 CHP models from the LGRC study to train our models and evaluated them on 103 IPF and 82 CHP samples from GSE150910. We again used the PRAUC scores to compare the models and computed their FDR-based significance statistics. All our experiments described in this section were repeated using three different gene sets (170 intramodular hubs, 103 novel candidates and 26 secreted proteins) identified in our study.

REFERENCES

1. Zhang, B. and S. Horvath, *A general framework for weighted gene co-expression network analysis.* Stat Appl Genet Mol Biol, 2005. **4**: p. Article17.
2. Langfelder, P. and S. Horvath, *Eigengene networks for studying the relationships between co-expression modules.* BMC Syst Biol, 2007. **1**: p. 54.
3. Langfelder, P., B. Zhang, and S. Horvath, *Defining clusters from a hierarchical cluster tree: the Dynamic Tree Cut package for R.* Bioinformatics, 2008. **24**(5): p. 719-20.
4. Langfelder, P., et al., *Is my network module preserved and reproducible?* PLoS Comput Biol, 2011. **7**(1): p. e1001057.
5. Horvath, S. and J. Dong, *Geometric interpretation of gene coexpression network analysis.* PLoS Comput Biol, 2008. **4**(8): p. e1000117.
6. Langfelder, P., P.S. Mischel, and S. Horvath, *When is hub gene selection better than standard meta-analysis?* PLoS One, 2013. **8**(4): p. e61505.
7. DePianto, D.J., et al., *Heterogeneous gene expression signatures correspond to distinct lung pathologies and biomarkers of disease severity in idiopathic pulmonary fibrosis.* Thorax, 2015. **70**(1): p. 48-56.
8. Sivakumar, P., et al., *RNA sequencing of transplant-stage idiopathic pulmonary fibrosis lung reveals unique pathway regulation.* ERJ Open Res, 2019. **5**(3).
9. Furusawa, H., et al., *Chronic Hypersensitivity Pneumonitis, an Interstitial Lung Disease with Distinct Molecular Signatures.* Am J Respir Crit Care Med, 2020. **202**(10): p. 1430-1444.
10. Langfelder, P. and S. Horvath, *WGCNA: an R package for weighted correlation network analysis.* BMC Bioinformatics, 2008. **9**: p. 559.
11. Friedman, J.H., T. Hastie, and R. Tibshirani, *Regularization Paths for Generalized Linear Models via Coordinate Descent.* Journal of Statistical Software; Vol 1, Issue 1 (2010), 2010.
